# Prevalence and risk factors for dyslipidemia among adults in rural and urban China: findings from the China National Stroke Screening and prevention project (CNSSPP)

**DOI:** 10.1186/s12889-019-7827-5

**Published:** 2019-11-11

**Authors:** Sampson Opoku, Yong Gan, Wenning Fu, Dajie Chen, Emmanuel Addo-Yobo, Diana Trofimovitch, Wei Yue, Feng Yan, Zhihong Wang, Zuxun Lu

**Affiliations:** 1 Department of Social Medicine and Health Management, School of Public Health, Tongji Medical College, Huazhong University of Science and Technology, No. 13 Hangkong Road, Wuhan, 430030 China; 2 Department of Internal Medicine, SUNY Upstate Medical University, New York, USA; 3 Department of Internal Medicine, East Tennessee State University, Johnson City, Tennessee USA; 4 Department of Neurology, Tianjin Huanhu Hospital, Tianjin, China; 5 Department of Neurosurgery, Xuanwu Hospital, Capital medical University, Beijing, China; 6 Department of Neurology, Shenzhen Second People’s Hospital, Shenzhen University, Shenzhen, Guangdong China

**Keywords:** Dyslipidemia, Rural, Urban, Risk factors, Prevalence

## Abstract

**Background:**

Dyslipidemia is a modifiable risk factor for cardiovascular disease (CVD). We investigated the prevalence and associated risk factors of dyslipidemia- raised total cholesterol (TC), raised triglycerides (TG), raised low-density lipoprotein (LDL-C), low high-density lipoprotein (HDL-C), and raised non-high-density lipoprotein (non-HDL-C) in rural and urban China.

**Methods:**

We analyzed data from 136,945 participants aged 40–100 years of the CNSSPP project for 2014. Dyslipidemia was defined by the NCEP-ATP III and the 2016 Chinese guidelines for the management of dyslipidemia in adults. Complete data on demographic, metabolic and lifestyle characteristics were used. Chi-square tests and multivariable logistic regression were used to obtain age- and sex-adjusted prevalence and risk factors for dyslipidemia among participants.

**Results:**

A total of 53.1% participants lived in rural areas. The prevalence of dyslipidemia was similar among rural and urban participants (43.2% vs. 43.3%). Regarding the components of dyslipidemia: urban compared with rural participants had a higher prevalence of low HDL-C (20.8% vs. 19.2%), whereas the prevalence of raised LDL-C (7.8% vs. 8.3%), raised TC (10.9% vs.11.8%) and raised non-HDL-C (10.0% vs. 10.9%) were lower in urban residents, (all *p* < 0.001). Women were more likely to have raised TC than men (adjusted OR [AOR] =1.83, 95% confidence interval [CI]:1.75–1.91), raised LDL-C (AOR = 1.55, 95% CI: 1.47–1.63) and high non-HDL-C (AOR = 1.52 95% CI: 1.45–1.59) (all *p* < 0.001). Compared with rural, urban participants had higher odds of dyslipidemia: low HDL-C (AOR = 1.04, 95% CI: 1.01–1.07), and raised TG (AOR = 1.06, 95% CI: 1.04–1.09). Hypertension and current drinker were less likely to get low HDL-C with AOR 0.93 (95% CI: 0.90–0.96) and AOR 0.73 (95% CI: 0.70–75), respectively. Overweight, obesity, central obesity and diabetes had higher odds of all dyslipidemias (*p* < 0.001).

**Conclusions:**

Low HDL-C was higher in urban areas, whereas the remaining dyslipidemia types were more common in rural areas**.** Dyslipidemia was more common in women in both areas of residence. Overweight, obesity, central obesity and diabetes were associated with dyslipidemias. The need to intensify intervention programs to manage dyslipidemia and risk factors should be prioritized.

## Background

Cardiovascular disease (CVD) constitutes the greatest cause of morbidity and mortality with a high incidence and prevalence in countries of all economic groups [1]. In China, CVD is a leading contributor to morbidity, mortality and disability-adjusted life-years (DALYs) [2]. In addition, an increased prevalence of CVD implies high costs to individuals and the state [3]. Therefore, managing the risk factors for CVD is necessary for the control of cardiovascular disease epidemic in China [4]. Prior studies in China and other countries have linked CVD events and burden to factors like poor knowledge of risk factors, changes in lifestyle due to rapid urbanization, epidemiologic and economic transitions [5–8].

Dyslipidemia is a major cardiovascular risk factor; it is a group of metabolic derangements characterized by any or a combination of the following: raised low-density lipoprotein cholesterol (LDL-C), raised total cholesterol (TC), raised triglycerides (TG) and low high-density lipoprotein cholesterol (HDL-C) [9].

Lipid abnormalities are associated with an increased risk of CV events including coronary heart disease (CHD), thereby serving as contributors to this process. The impact of cholesterol abnormalities on CHD risk is increased if other risk factors such as obesity are simultaneously present. Adverse lipid profiles have been found to be a risk factor for the first coronary event. It appears to be highly predictive of recurrent events, too [10–12]. Therefore, the prevention and control of dyslipidemia especially in adult populations are important to help reduce CV events.

China has experienced dramatic socio-economic transition in the past decades with great rural-urban migration [13, 14]. Earlier studies in parts of China have shown trends towards increase in the prevalence of dyslipidemia in urban areas [6, 15]. However, limited data are available on the nationwide distribution of dyslipidemia prevalence based on rural vs. urban living. It is possible that those who live in rural areas may have a less healthy lifestyle and therefore could have higher prevalence [16]. Therefore, we aimed to determine the prevalence of dyslipidemia and risk factors among adults aged ≥40 living in rural and urban China.

## Methods

### Study population

This study utilized the 2014 data from the China National Stroke Screening and Prevention Project (CNSSPP). It was a survey conducted by the National Project Office for Stroke Prevention and Control that consists of a representative sample of the Chinese adult’s population. Details of this survey have been described elsewhere [17]. Briefly, it was a cross-sectional survey involving Chinese adults aged 40 years or older. Employing a multistage cluster probability sampling strategy, 200 project areas were first selected in proportion to the local population size and the total number of counties. Subsequently, an urban community and a rural village were selected from each project area as primary sampling units according to geographical locations and suggestions from local hospitals. The cluster sampling method was used in every primary sampling unit and all residents aged 40 years or above were interviewed during the primary screening sections.

### Measurement of indicators

A questionnaire was used to obtain baseline information from participants by trained investigators. It contained demographic characteristics (such as age, sex, and ethnicity), geographical zones (such as north and south, and stroke belt), medical history (such as CHD, dyslipidemia, stroke, hypertension and diabetes), and lifestyle activities (such as tobacco smoking, alcohol use and physical activity). The baseline data were further categorized by other parameters. Age was recorded as subjects’ current age and grouped as follows: ‘40–49′, ‘50–59′, ‘60–69′, and ‘70’ years and above [15]. Smoking status was classified into ‘non-smoker’ and ‘current smoker’ (current smoker is one who smokes at least one cigarette per day). Alcohol use status was categorized into ‘current drinker’ and ‘non-drinker’ (current drinker is one who drinks alcoholic beverages ≥1 per week for more than half a year). Physical activity was classified into ‘yes’ and ‘no’ (yes refers to physical activity of ≥3 times per week for at least 30 min each episode, or engaged in heavy physical work) by self-reported information.

Nationality was grouped into two: Han and other Ethnicities. Socio-economic regions were classified as high, middle, and low-income levels according to the per capita disposable income household’s thresholds in 2014 [18]. High-income regions included Beijing, Fujian, Guangdong, Jiangsu, Liaoning, Inner Mongolia, Shanghai, Shandong, Tianjin and Zhejiang. Middle-income regions were Anhui, Chongqing, Hainan, Hebei, Heilongjiang, Hubei, Hunan, Jilin, Jiangxi and Shanxi. And low-income regions included Henan, Guangxi, Sichuan, Guizhou, Yunnan, Shaanxi, Gansu, Qinghai, Ningxia, and Xinjiang.

### Definitions

An urban area was defined with a 12-component study-specific urbanization index. This criterion had been previously validated to capture the degree of urbanization in the study communities (reliability across study waves [Cronbach’s Alpha]: 0.85–0.89; validity [correlation with official classification]; 0.75–0.78) [19]. The following 12 components were included in the development of the urbanization index: (1) population density, (2) housing, (3) traditional market, (4) sanitation, (5) transportation and infrastructure, (6) modern markets, (7) communications (e.g. TV and cinema), (8) types of economic activity (e.g. electricity and flush toilet), (9) education, (10) diversity (i.e. greater diversity in community education level, and greater variation in community income level), (11) health infrastructure, and (12) social services such as the provision of preschool for children under 3 years old, availability of commercial medical insurance, free medical insurance, and/or insurance for women and children. The scale has a range of 0–120, the higher the score, the more the urban characteristics across these twelve multiple domains.

Geographically, we classified all participants into two zones: 1. the northern and southern zones according to the Huai River–Qin Mountains Line. The Northern zone included: Heilongjiang, Jilin, Liaoning, Inner Mongolia, Beijing, Tianjing, Hebei, Henan, Anhui, Shandong, Shanxi, Shaanxi, Gansu, Ningxia, Qinghai and Xinjiang. The southern part included: Jiangsu, Zhejiang, Shanghai, Chongqing, Sichuan, Fujian, Jiangxi, Hubei, Hunan, Guizhou, Guangzhou, Guangxi, Hainan and Yunnan [20]. 2. The stroke belt. The stroke belt was defined based on a region containing provinces that met the following two (2) criteria for a region of high stroke incidence: (1) the province was ranked in the top one third of provinces for stroke incidence (i.e., met the above criteria for high stroke incidence) and (2) more than one third (> 33.3%) of the prefectural regions within the province were ranked in the top two sevenths of prefectural regions for stroke incidence (i.e. met the above criteria for high stroke incidence) [21].

A history of CHD, dyslipidemia and stroke was defined as self-report of any previous diagnosis of CHD, dyslipidemia and stroke by a healthcare professional or currently undergoing treatment. Hypertension was diagnosed if the subject had a history of hypertension, or a systolic blood pressure (SBP) ≥ 140 mmHg, or a diastolic blood pressure ≥ 90 mmHg, or currently taking antihypertensive medications. Diabetes mellitus was diagnosed if the subject had a history of diabetes mellitus, or currently undergoing treatment with insulin or oral hypoglycemic agents, or the fasting blood glucose (FBG) level was ≥7.0 mmol/L.

### Physical examination

Body weight and height were measured and then body mass index (BMI) was calculated as body weight (kg) divided by the square of height (m^2^) based on the Criteria of the Ministry of Health, China [22]. Individuals were categorized into four groups: BMI < 18.5 kg/m^2^ (underweight), ≥ 18.5 kg/m^2^ and < 24 kg/m^2^ (normal weight), ≥ 24 kg/m^2^ and < 28 kg/m^2^ (overweight), and ≥ 28 kg/m^2^ (obesity). Waist circumference (WC) was measured at the midpoint between the iliac crest and the lower rib. Men with waist circumference ≥ 90 cm or women with waist circumference ≥ 85 cm were defined as central obesity. Blood pressure of participants was measured after a 20-min seated rest and the mean of three measurements was used for this study.

### Biochemical measurements

A standardized protocol was adopted for blood collection and used at all research centers. Only laboratories that could perform all the biochemical analyses of the study with strict quality assurance to receive and manage the collection of samples were used. This allowed for measures to be standardized and results to be made uniform. All laboratories received instructions on the protocol to be followed including labelling of kits for blood collection for each adult. Blood samples were obtained from subjects in the morning after fasting for at least 8 h, and were examined for FBG, TC, TG, LDL-C and HDL-C according to standard protocols.

Lipid abnormalities were classified according to the Third Report of the National Cholesterol Education Program (NCEP) Expert Panel on Detection, Evaluation, and Treatment of High Blood Cholesterol in Adults final report (NCEP-ATP III) [23], these classifications were same as the criteria of the 2016 Chinese guidelines for the management of dyslipidemia in adults [10]. Borderline high TC level: ≥ 5.22 and < 6.22 mmol/L, high TC level: ≥ 6.22 mmol/L, borderline high LDL-C: ≥ 3.44 and < 4.1 mmol/L, high LDL-C: ≥ 4.14 mmol/L, low HDL-C: < 1.04 mmol/L, borderline high TG: ≥ 1.7 and < 2.26 mmol/L, and elevated TG: ≥ 2.26 mmol/L. Non- HDL-C was calculated as TC minus HDL-C. Borderline high non-HDL-C was defined as ≥4.1 and < 4.9 mmol/L, and high non-HDL-C was defined as ≥4.9 mmol/L [24].

### Statistical analysis

Demographic, metabolic and lifestyle characteristics of participants by residence (rural versus urban) were expressed using descriptive statistics. The normality of the continuous variables was assessed, and variables with a skewed distribution were reported with medians and interquartile ranges (IQRs), and the chi- square test was used for the categorical variables. The prevalence and 95% confidence interval (95%CI) for the overall and components of dyslipidemia by sex, age group and area of residence were calculated according to the 2010 population census age and sex distribution of China.

Multivariable logistic regression model was used to analyse the risk factors. In the multivariable analysis, participants in the socio-economic regions were compared with low-income region and normal, underweight, overweight and obese were compared to normal weight participants. Statistical significance was accepted at the 5% level (*p* < 0.05). All statistical analyses were performed using SPSS 19.0 (SPSS Inc., Chicago, Ill). Furthermore, processing of graphs and tables was done using MS Excel 2013.

## Results

### Population description

A total of 726,451 Chinese adults were recorded in the 2014/2015 CNSSPP database. This study focused on 180,000 subjects randomly selected from all provinces and municipalities in china. These people were selected for further assessment for stroke risk factors including high blood pressure, abnormal serum lipids and physical examinations [25]. Our analysis was based on the 136,945 (19%) weighted subjects between 40 and 100 years, with complete demographic, metabolic and lifestyle data.

More than half of the respondents were from rural areas 53.1% (*n* = 72,712). Most of the subjects from both rural and urban settings were Hans (96.7%). The age- and sex- specific median (IQR) of participants in rural and urban areas were 55.0 (49.0–62.0) and 56 (50.0–63.0), respectively. Urban areas recorded more subjects with history of CHD, dyslipidemia, stroke and come from the stroke belt zone.

The largest proportion of respondents were between the ages of 50 to 59 years: 41.8% (*n* = 30,366) from rural areas, and 42.8% (*n* = 27,494) from urban areas. Compared with urban participants, rural ones were more likely to have hypertension, 63.5% (*n* = 46,155) vs. 61.2% (*n* = 39,280); smoke, 35.3% (*n* = 25,677) vs. 29.9% (*n* = 19,174); and be from low and middle- income regions, (28.0% [*n* = 20,395]) vs. (18.5% [*n* = 11,909]), and (32.8% [*n* = 23,853] vs. (26.6% [*n* = 17,097]); *p* value from χ^2^ < 0.001). They were also more likely to have higher median lipid values. For example, 4.92 [4.22–5.64]) vs. 4.88 [4.23–5.58]) for TC. Urban compared to rural residents were more likely to be obese, physically inactive and to have diabetes (*p* value from χ^2^ < 0.001) (Table 1).

**Table 1:** Demographic, metabolic and lifestyle characteristics of rural and urban 40–100 year-old Chinese adults

	Rural (*N* = 72,712)	Urban (*N* = 64,233)	Total (*N* = 13,6945)
	No. (%)	No. (%)	No. (%)
Age			
Median (IQR)	55.0 (49.0–62.0)	56.0 (50.0–63.0)	55.0 (49.0–63.0)
40–49	20,129 (27.7)	15,922 (24.8)	36,051 (26.3)
50–59	30,366 (41.8)	27,494 (42.8)	57,860 (42.3)
60–69	13,923 (19.1)	12,560 (19.6)	26,483 (19.3)
70 and above	8294 (11.4)	8257 (12.8)	16,552 (12.1)
Sex			
Men	37,728 (51.9)	32,835 (51.1)	70,563 (51.5)
Women	34,984 (48.1)	31,398 (48.9)	66,382 (48.5)
History of CHD			
Yes	65,704 (90.4)	58,702 (91.4)	12,4406 (90.8)
No	7008 (9.6)	5531 (8.6)	12,539 (9.2)
History of Dyslipidemia			
Yes	44,834 (61.8)	40,346 (64.2)	85,180 (62.9)
No	27,722 (38.2)	22,501 (35.8)	50,223 (37.1)
History of Stroke			
Yes	56,563 (77.8)	51,846 (80.7)	10,8409 (79.2)
No	16,150 (22.2)	12,387 (19.3)	28,537 (20.8)
Geographical Regions			
^b^North	39,102 (53.8)	37,093 (57.7)	76,195 (55.6)
South	33,611 (46.2)	27,140 (42.3)	60,751 (44.4)
^c^Stroke Belt	11,960 (16.4)	11,689 (18.2)	23,649 (17.3)
Non-stroke Belt	60,753 (83.6)	52,544 (81.8)	11,3297(82.7)
Socio- economic region			
Low- income	20,395 (28.0)	11,909 (18.5)	32,304 (23.6)
Middle- income	23,853 (32.8)	17,097 (26.6)	40,950 (29.9)
High-income	28,464 (39.1)	35,223 (54.8)	63,691 (46.5)
Han Nationality	70,333 (96.7)	62,106 (96.7)	13,2439 (96.7)
BMI^a^			
Median (IQR)	25.0 (22.8–27.6)	25.3 (23.2–27.4)	25.2 (23.0–27.5)
Underweight (< 18.5 kg/m2	1050 (1.4)	802 (1.2)	1852 (1.4)
Normal (18.5 - < 24.0)	25,864 (35.6)	21,237 (33.1)	47,101 (34.4)
Overweight (24.0 - < 28)	30,690 (42.2)	29,316 (45.6)	60,006 (43.8)
Obesity (≥28.0)	15,108 (20.8)	12,879 (20.1)	27,987 (20.4)
Waist circumference^†^			
Median (IQR)	86.0 (80.0–93.0)	86.0 (80.0–93.0)	86.0 (80.0–93.0)
Normal < 90/85 cm (M/W)	31,077 (42.7)	25,676 (40.9)	56,753 (41.9)
Central obese ≥90/85 cm (M/W)	41,621 (57.3)	37,083 (59.1)	78,704 (58.1)
Diabetes Mellitus	20,529 (28.2)	21,008 (32.7)	41,537 (30.3)
Hypertension	46,155 (63.5)	39,280 (61.2)	85,435 (62.4)
Drinking (Current)	14,082 (19.4)	12,084 (18.8)	26,166 (19.1)
Smoking (Current)	25,677 (35.3)	19,174 (29.9)	44,851 (32.8)
Physical inactivity	23,455 (32.3)	28,599 (44.5)	52,054 (38.0)
Lipid variables: Median (IQR)			
TC mmol/l	4.92 (4.22–5.64)	4.88 (4.23–5.58)	4.90 (4.23–5.61)
HDL-C mmol/l	1.35 (1.10–1.65)	1.28 (1.08–1.55)	1.31 (1.09–1.60)
LDL-C mmol/l	2.85 (2.20–3.42)	2.85 (2.24–3.40)	2.85 (2.21–3.41)
Triglycerides mmol/l	1.50 (1.04–2.16)	1.51 (1.09–2.15)	1.50 (1.06–2.15)
Non-HDL-C^‡^ mmol/l	3.51 (2.80–4.24)	3.53 (2.88–4.22)	3.52 (2.84–4.23)

Data are N (%) or median (IQR), interquartile range. Waist circumference^†^ has missing values of 1489 (1.1%);

Non-HDL-C^‡^ has missing values of 76 (0.1%). *TC* total cholesterol; *HDL-C* high-density lipoprotein; *LDL-C* low-density lipoprotein: triglycerides; *M/W* Men/ Women; BMI^a^ calculated as weight in kilograms divided by height in meters squared: ^b,c^ Two different geographic zones of china

The serum lipid concentrations of the study population by area of residence.

Table 2 summarizes the percentages (with 95% confidence intervals) of serum lipid concentrations of the study participants according to the area of residence. The overall adjusted prevalence of participants with borderline-high TC was 28.0% (95% CI 27.8–28.2); high TC: 11.3% (95% CI 11.2–11.5); borderline-high LDL-C: 18.4% (95% CI 18.2–18.6); and high LDL-C: 8.1% (95% CI 7.9–8.2). Rural participants were more likely to have high LDL-C 8.3% (95% CI 8.1–8.5), than their urban counterparts 7.8% (95% CI 7.5–8.0). Urban versus rural residents were more likely to get low HDL-C levels 20.8% (95% CI 20.5–21.1) vs. 19.2 (95% CI 18.9–19.5).

**Table 2:** The serum lipid concentrations of the study population by area of residence

	Overall	Rural	Urban
LIPID VARIABLES	P (95% CI)	P (95% CI)	P (95% CI)
Total cholesterol mmol/L (mg/dL)			
Desirable <5.2 (< 200)	60.7 (60.4–60.9)	59.5 (59.2–59.9)	61.9 (61.5–62.3)
Borderline high ≥5.2 (200) and - < 6.2 (240)	28 .0 (27.8–28.2)	28.7 (28.3–29.0)	27.2 (26.9–27.6)
High ≥6.22 (240)	11.3 (11.2–11.5)	11.8 (11.6–12.0)	10.9 (10.6–11.1)
LDL-C* mmol/L (mg/dL)			
Desirable < 2.6 (100)	39.1 (38.8–38.3)	39.2 (38.9–39.6)	38.9 (38.5–39.2)
Near optimal ≥2.6 (100) and < 3.4 (130)	34.5 (34.2–34.7)	34 (33.6–34.3)	35 (34.6–35.3)
Borderline high ≥3.4 (130) and 4.1(160)	18.4 (18.2–18.6)	18.4 18.1–18.7)	18.4 18.1–18.7)
High ≥4.1 (160)	8.1 (7.9–8.2)	8.3 (8.1–8.5)	7.8 (7.6–8.0)
HDL-C* mmol/L (mg/dL)			
Low < 1.0 (< 40)	19.9 (19.7–20.1)	19.2 (18.9–19.5)	20.8 (20.5–21.1)
Desirable ≥1.0 (40) and 1.6 (60)	51.6 (51.3–51.9)	48.9 48.6–49.3)	54.7 (54.3–55.0)
High ≥1.6(≥60)	28.5 (28.2–28.7)	31.9 (31.6–32.2)	24.6 (24.2–24.9)
Triglycerides, mmol/L (mg/dL)			
Desirable < 1.7 (< 150)	58.5 (58.2–58.7)	58.4 (58.1–58.8)	58.5 (58.2–58.9)
Borderline high 1.7 (150) and 2.3 (200)	19.1 (18.9–19.3)	19.2 (18.9–19.5)	19 (18.7–19.3)
High ≥2.3 (≥200)	22.4 (22.2–22.6)	22.4 (22.1–22.7)	22.5 (22. 2–22.8)
Non- HDL-C* mmo/l (mg/dl)			
Desirable < 4.1 (< 160)	70.9 (70.6–71.1)	70.8 (70.4–71.1)	71 (70.6–71.3)
Borderline ≥4.1 (160) and < 4.9 (190)	18.7 (18.5–18.9)	18.3 (18.1–18.6)	19 (18.7–19.3)
High ≥4.9 (190)	10.5 (10.3–10.6)	10.9 (10.7–11.1)	10 (9.8–10.2)

*LDL-C low-density lipoprotein; *HDL-C high-density lipoprotein. †Non HDL-C has missing values of 76

(0.1%); *P* percentage; *CI* Confidence interval

### Prevalence of dyslipidemia in rural and urban areas

**Fig. 1:**
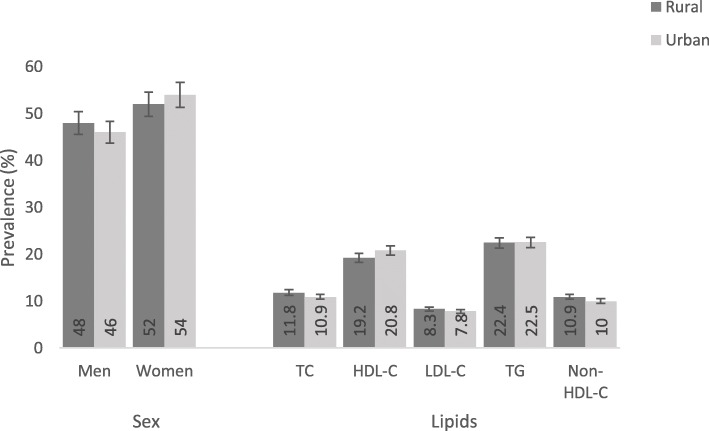
Prevalence of dyslipidemia according to area of residence among the study population

The age- and sex- standardized prevalence of dyslipidemia was 43%. The prevalence of dyslipidemia was similar among rural and urban participants (43.2% vs. 43.3%), (figure not shown). As presented in Fig. 1, compared with men, women had higher prevalence of dyslipidemia in both areas of residence: (rural: 52.0% vs. 48.0%, urban: 54.0% vs. 46.0%; *p* for χ^2^ < 0.001). Regarding the five components of dyslipidemia, low HDL-C recorded higher prevalence in urban as compared to rural participants (20.8% vs. 19.2%), whereas raised LDL-C had higher prevalence among rural compared to urban populations (8.3% vs. 7.8%; (*p* value from χ^2^ < 0.001). Raised TC and raised non-HDL-C also had higher prevalence in rural compared with urban subjects (11.8% vs.10.9 and 10.9% vs. 10.0%; *p* for χ^2^ < 0.001), respectively. Elevated TG prevalence among rural and urban adults was comparable (22.4% vs. 22.5%).

**Fig. 2:**
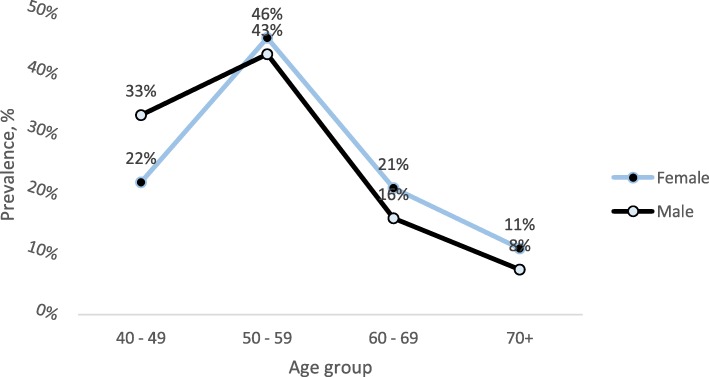
Prevalence of dyslipidemia by different age groups and sex among the study population

Regarding the prevalence of dyslipidemia stratified by sex and age groups, significant differences were observed. Men below age 50 had a higher prevalence of dyslipidemia (33%) than women (22%). The peak prevalence of dyslipidemia in both sexes converged at aged 50–59 years. Afterwards, prevalence declined simultaneously in both sexes, with women recording higher values than men (*p* < 0.05) (Fig. 2).

**Fig. 3:**
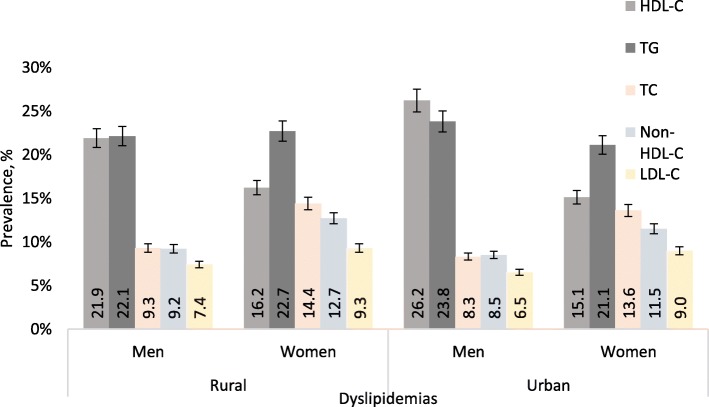
Prevalence of dyslipidemia according to sex in rural and urban residences among the study population

Fig. 3 shows significant differences in dyslipidemia components according to sex in both rural and urban settings. All lipid abnormalities were significantly higher among women than among men in rural settings (*p* for χ^2^ < 0.001) except low HDL-C. Similarly, urban women had higher levels for raised TC, raised LDL-C and raised non-HDL-C (*p* for χ^2^ < 0.001). While, low HDL-C and raised TG levels were higher among urban men compared with women (26.2% vs. 16.5 and 23.8% vs. 21.1%; *p* for χ^2^ < 0.001), respectively.

**Fig. 4:**
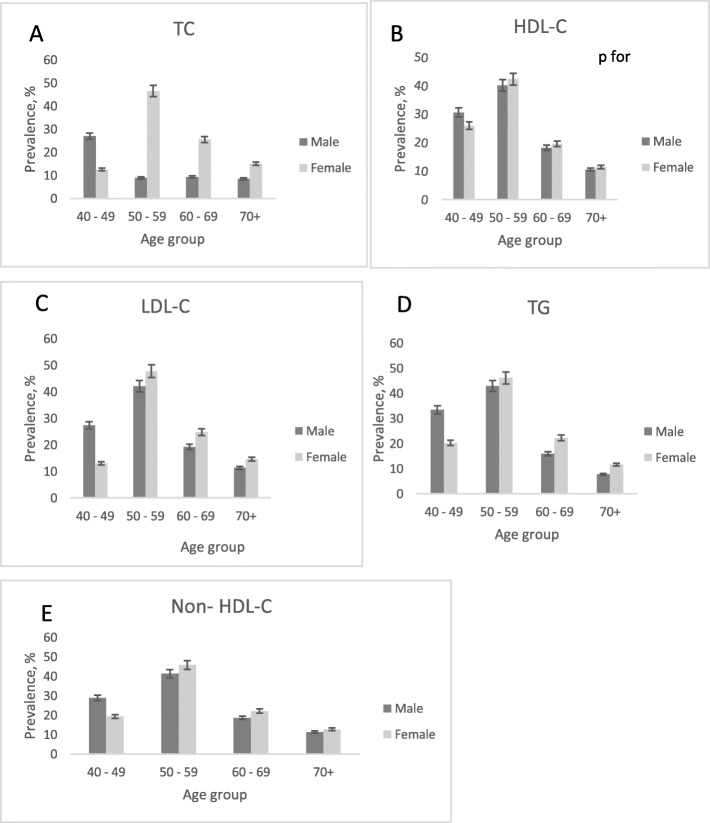
**a**-**e**: Age- and sex-specific prevalence of dyslipidemia components in the study

Figure 4 presents the prevalence of the five components of dyslipidemia according to sex and age groups. Similar prevalence patterns were observed among all the components. The patterns increased and decreased with ageing, and were significantly different between men and women (*p* value from χ^2^ < 0.001). The prevalence peaked at aged 50–59 years and declined afterwards. Below age 50, men were more likely than women to have dyslipidemia, whereas after age 59, men showed lower risk of dyslipidemia than women.

### Risk factors associated with dyslipidemia

Table 3 describes the age- and sex- adjusted prevalence of dyslipidemia stratified by CVD risk factors**.** Men versus women had higher raised TC, low HDL-C and raised TG values (14% vs. 9, 24% vs. 16 and 23% vs. 22%), respectively. A higher prevalence of dyslipidemia was observed in obese and diabetic participants. Raised TG was 23% in smokers and 26% in drinkers.

**Table 3:** Prevalence of dyslipidemia by some cardiovascular risk factors

	TC	HDL-C	LDL-C	TG	Non-HDL-C
Risk factors (N)	(%)	(%)	(%)	(%)	(%)
Male (70,563)	14	24	7	23	9
Female (66,383)	9	16	9	22	12
Normal/underweight (48,953)	10	17	7	16	9
Overweight (60,006)	11	21	8	24	11
Obese (27,986)	13	23	10	30	13
Diabetic (95,407)	13	22	9	26	12
Non- diabetic (41,537)	11	19	8	21	10
Hypertensive (85,435)	13	20	9	25	12
Non-hypertensive (51,510)	10	20	7	19	9
Smoker (44,851)	10	23	8	23	9
Non-Smoker (92,095)	12	19	8	22	11
Drinker (26,166)	11	20	8	26	10
Non-drinker (11,0778)	11	20	8	22	11
Physical activity (52,054)	12	20	9	24	11
Physical inactivity (84,891)	11	20	8	22	10

*LDL-C* raised low-density lipoprotein; *HDL-C* low high-density lipoprotein; *TG* raised triglycerides *TC* raised total cholesterol; *N* total number of subjects

In the multivariable logistic regression analyses, subjects aged 50 years and above were more likely to have raised TC, raised LDL-C and raised non- HDL-C, but less likely to develop low HDL-C and raised TG than those aged 40–49 years. Women were at a higher risk of raised TC, raised LDL-C and raised non-HDL-C, but at a lower risk of low HDL-C than men. Urban residence, Han ethnicity and history of stroke showed significant associations with lipid abnormalities. History of dyslipidemia was a positively associated factor for dyslipidemia components except raised TG. Respondents with a history of CHD were unlikely to get dyslipidemia except raised TC. Overweight, obesity, central obesity and diabetes were associated with a high risk of all components of dyslipidemia. Geographically, people who stayed in the stroke belt and the northern parts of China had significant associations with raised TC, low HDL-C, raised LDL-C and raised TG. Hypertensives and current alcohol users were more likely to get all types of dyslipidemias except low HDL-C. Participants who engaged in regular physical activities were at a lesser risk of adverse blood lipid levels (Table 4).

**Table 4:** The associations of socio-demographic characteristics and cardiovascular disease risk factors with dyslipidemia in Chinese adults ≥40 years

	TC	HDL-C	LDL-C	TG	Non-HDL-C
Age (years)					
40–49	1.00	1.00	1.00	1.00	1.00
50–59	1.48 (1.41–1.55)	0.86 (0.83–0.89)	1.42 (1.35–1.50)	0.95 (0.92–0.98)	1.41 (1.34–1.47)
60–69	1.70 (1.61–1.80)	0.85 (0.81 - -0.88)	1.53 (1.44–1.63)	0.84 (0.81–0.87)	1.56 (1.47–1.64)
70 and above	1.64 (1.55–1.75)	0.77 (0.74–0.81)	1.49 (1.39–1.60)	0.68 (0.65–0.71)	1.49 (1.40–1.59)
Residence					
Rural	1.00	1.00	1.00	1.00	1.00
Urban	0.90 90.87–0.93)	1.04 (1.01–1.07)^C^	0.91 (0.87–0.94)	1.06 (1.04–1.09)	0.90 (0.87–0.94)
Sex					
Men	1.00	1.00	1.00	1.00	1.00
Women	1.83 (1.75–1.91)	0.62 (0.60–0.64)	1.55 (1.47–1.63)	1.02 (1.00 - 1.06)^NS^	1.52 (1.45–1.59)
Socio- economic regions					
Low-income	1.00	1.00	1.00	1.00	1.00
Middle-income	1.10 (1.05–1.16)	0.85 (0.82–0.88)	0.88 (0.83–0.93)	1.35 (1.30–1.40)	1.02 (0.98–1.08)^NS^
High-income	1.06 (1.01–1.11)^C^	0.82 (0.79–0.85)	0.90 (0.85–0.95)	0.92 (0.88–0.95)	0.97 (0.93–1.03)^NS^
Ethnicities					
Han nationality	1.76 (1.56–1.99)	0.71 (0.67–0.76)	0.88 (0.79–0.98)^C^	1.14 (1.06–1.22)^C^	1.50 (1.34–1.68)
Others	1.00	1.00	1.00	1.00	1.00
BMI					
Underweight	0.93 (0.78–1.09) ^NS^	0.87 (0.76–0.99)^C^	0.87 (0.71–1.06)^NS^	0.65 (0.56–0.75)	0.84 (0.71–1.01)^NS^
Normal weight	1.00	1.00	1.00	1.00	1.00
Overweight	1.11 (1.07–1.16)	1.15 (1.12–1.19)	1.18 (1.12–1.24)	1.41 (1.37–1.46)	1.21 (1.16–1.26)
Obesity	1.27 (1.20–1.33)	1.27 (1.22–1.33)	1.34 (1.26–1.42)	1.79 (1.72–1.87)	1.41 (1.33–1.48)
Central obesity					
Yes	1.05 (1.01–1.09)	1.24 (1,20–1 28)	1.13 (1.08–1.18)	1.25 (1.22–1.29)	1.08 (1.04–1.13)
No	1.00	1.00	1.00	1.00	1.00
History of CHD					
Yes	1.00 (0.94–1.06)^NS^	0.63 (0.60–0.66)	0.89 (0.83–0.96)^C^	0.77 (0,74–0.80)	0.91 (0.85–0.96)
No	1.00	1.00	1.00	1.00	1.00
History of Dyslipidemia					
Yes	1.06 (1.02–1.10)	1.09 (1.06–1.13)	1.18 (1.13–1.23)	0.98 (0.96–1.01)^NS^	1.09 (1.05–1.13)
No	1.00	1.00	1.00	1.00	1.00
History of Stroke					
Yes	0.92 (0.88–0.96)	0.93 (0.90–0.96)	1.00 (0.95–1.05)	0.73 (0.70–0.75)	0.91 (0.87–0.95)
No	1.00	1.00	1.00	1.00	1.00
Stroke Belt					
Yes	1.00 (0.95–1.05)	1.12 (1.07–1.17)	0.92 (0.86–0.97)^C^	1.44 (1.38–1.50)	0.99 (0.94–1.05)^NS^
No	1.00	1.00	1.00	1.00	1.00
Geographical region					
North	1.12 (1.07–1.17)	0.61(0.59–0.63)	1.13 (1.08–1.19)	0.67 (0.65–0.69)	0.98 (0.94–1.02)^NS^
South	1.00	1.00	1.00	1.00	1.00
Diabetes Mellitus					
Yes	1.14 (1.10–1.19)	1.18 (1.14–1.21)	1.17 (1.12–1.22)	1.25 (1.22–1.29)	1.21 (1.16–1.25)
No	1.00	1.00	1.00	1.00	1.00
Hypertension					
Yes	1.20 (1.15–1.24)	0.95 (0.92–0.97)	1.23 (1.18–1.29)	1.23 (1.19–1.26)	1.20 (1.15–1.24)
No	1.00	1.00	1.00	1.00	1.00
Current smoker					
Yes	1.02 (0.97–1.07)^NS^	1.01 (0.97–1.04) ^NS^	1.21 (1.15–1.28)	0.99 (0.96–1.03)^NS^	1.06 (1.01–1.12)^C^
No	1.00	1.00	1.00	1.00	1.00
Current drinker					
Yes	1.33 (1.27–1.40)	0.76 (0.73–0.79)	1.15 (1.08–1.22)	1.18 (1.13–1.22)	1.16 (1.10–1.22)
No	1.00	1.00	1.00	1.00	1.00
Physical activity					
Yes	0.96 (0.92–0.99)^C^	0.95 (0.92–0.97)	0.94 (0.90–0.98)^C^	0.89 (0.86–0.91)	0.96 (0.92–0.99)^C^
No	1.00	1.00	1.00	1.00	1.00

Figures are both locations pooled ORs (95% CIs). 1.00 = reference group

All the presented 0Rs are significant at *p* < 0.001 except for: ^*C*^*p* < 0.05; NS, not significant (*p* > 0.05)

CNSSPP database, *HDL-C* low high-density lipoprotein; *LDL-C* raised low-density lipoprotein; *TG* raised triglycerides, *TC* raised total cholesterol; *Non- HDL-C* raised non-high-density lipoprotein

## Discussion

Our study used a large population of Chinese adults of 40 years or above, who were enrolled in the 2014 CNSSPP. To our knowledge, this is the first study to nationally compare rural and urban prevalence of dyslipidemia and risk factors among middle-aged and older Chinese adults (≥40 years). The study participants were all resident in China, having been selected randomly from rural and urban areas of all provinces and municipalities of the country except Tibet. Therefore, this study offers a representative feature applicable to the Chinese population.

### Main findings

The following were the main findings of this study: first, the overall adjusted prevalence of dyslipidemia was 43%. The overall dyslipidemia prevalence in rural and urban areas were comparable, 43.2 and 43.3%, respectively. Further, our data suggested that the adjusted prevalence of low HDL-C was higher in urban than in rural residents. Raised LDL-C, raised TC and raised non-HDL-C levels were higher among rural than among urban residents and the difference was more pronounced in raised TC. Additionally, we identified overweight, obesity, central obesity, and diabetes as positive associated factors of dyslipidemias. Age groups, urban residence, Han nationality, current alcohol use, and hypertension showed significant associations with all dyslipidemias types. Regular physical activity was negatively associated with dyslipidemia.

### Comparison of findings with other studies

#### Prevalence of dyslipidemia

In the past few decades, CVD has increased in low- and middle-income countries with a decline in several developed countries [26]. In view of this, it is crucial to understand the role of major risk factors of this emerging epidemic, dyslipidemia being one of them, and their effective control. The overall dyslipidemia prevalence (43%) found in this study was higher than the 34.0% reported by a recent study [27], and 41.9% by a study of dyslipidemia prevalence among Chinese adults in 2014 [28]. Likewise, the overall prevalence of dyslipidemia in rural and urban areas of our study (as indicated earlier), was higher than the rural (17.7%) and urban (21%) prevalence reported among Chinese adults in 2005 [29]. The low HDL-C level observed among urban residents was consistent with earlier reports [30–32], and showed the unhealthy HDL-C lipid profile status among urban adults which could put them at risk of coronary artery disease (CAD) [11]. The high prevalence of raised TC, raised non-HDL-C and raised LDL-C levels among rural residents was similar to findings from a previous study among adults in rural areas of Northern China [33]. The reasons for the above results could be attributed to the following: first, the dramatic epidemiologic transition (i.e. the shift from infectious to chronic diseases) experienced by China in recent years. This shift was driven by increases in the prevalence of many known risk factors for chronic diseases such as unhealthy lifestyles (e.g. decreased physical activity, excessive smoking, excessive alcohol use, and westernized diet) [34, 35]. Second, is the impact of China’s rapid economic growth and urbanization. A study has noted that poor early life conditions could increase the risk of obesity in a subsequently more socio-economically developed environment [36]. Again, to some extent, urbanized rural villages might share the environmental risk factors of cities (e.g. water and air pollutions), but may not enjoyed the advantage (e.g. available recreational resources, access to convenient health care services). And our study found that subjects from rural areas had higher values for current drinking, current smoking and hypertension. This result was consistent with a previous report which indicated that current smoking and drinking increase the risk of dyslipidemia [37]. A study in Central India reported a higher dyslipidemia prevalence in rural (74.5%) and urban (68.8%) areas [38]. The differences in prevalence between our study and the Indian study could be attributed to the disparities in lifestyles, genetics and socio-economic status, as well as the diagnostic criteria used in these studies. The situation has important policy implications for health service planners and policy makers in China to design appropriate health programs and strategies to control this disease.

The prevalence of raised TG as found in our study was high and similar in both living areas- rural (22.4%) and urban (22.5%). This suggests that the included populations could be prone to CHD [12]. Elevated TG levels have been identified as a residual CV risk, even in persons on LDL-C lowering therapies like statins [39, 40]. As a result, the adoption and promotion of healthy lifestyle among adults in addition to the usage of TG-lowering agents could help reduce this abnormality and residual risks [41].

#### Risk factors associated with dyslipidemia

Age, according to the ATP III protocol, is a known non-modifiable risk factor for CHD, and CHD risk increases sharply with advancing age [42]. In our study, adults of all age groups had positive associations with raised TC, raised LDL-C and raised non- HDL-C, which was partially consistent with a previous study [10]. At any given level of LDL-C, the risk for CHD is higher in older than in younger people. Also, TC level increases with ageing, but remains relatively stable around age 70 [10]. Some earlier studies have found that TG and other lipoprotein levels could increase as a result of increasing age [10, 13, 43]. Due to the ongoing demographic transition in China, the proportion of people of advancing age is also expected to go up [14]. With increasing age as a predisposing factor for dyslipidemia, the elderly are at a greater risk of developing CVD. Therefore, preventive strategies including regular screening of serum lipids and initiation of early treatments among older people should be intensified.

One study proved that at any given age, men are at greater risk for CHD compared with women. Additionally, the male sex was considered as a non-modifiable risk factor for CHD by the ATP III guidelines [42]. It was therefore unsurprising from our results that women had lower odds of low HDL-C. Nonetheless, the underlying etiologies for this observed gender disparity regarding the likelihood of developing CHD are not completely understood. One probable explanation is the earlier onset of CHD risk factors such as hypertension in men. However, the Framingham Heart Study noted that absolute risk differences in both sexes cannot be entirely explained by standard risk factors [42]. For this reason, further studies are required with regard to pathophysiological, behavioral, and socio-demographic risk factors that could contribute to this higher prevalence of low HDL-C observed among men. However, women demonstrated higher likelihoods for the following lipid abnormalities in our study: raised TC, raised non-HDL-C and raised LDL-C. This finding was comparable to an earlier study which noted that menopause can affect lipid parameters by increasing LDL-C, TG and TC levels and decreasing HDL-C level. Considering the ages of the women in this study, some of them could be in postmenopausal state. Hence, we speculate that these differences could be as a result of the effects of sex hormones [44].

In the present study, we also analyzed the associations between the individual components of dyslipidemia and the personal histories of CHD, dyslipidemia and stroke. We found that personal history of stroke was positively associated with raised LDL-C. The personal history of dyslipidemia was associated with higher odds of raised (TC, LDL-C, non-HDL-C), and low HDL-C. These observed associations support the beneficial health effects of effective control strategies such as awareness creation [45], regular medical screening and monitoring of serum lipids among adults for the prevention and management of dyslipidemia. Several potential mechanisms might have contributed to these relationships hence, further investigations would be required to explain the findings.

Participants from the northern zone of China demonstrated significant associations with raised TC and raised LDL-C. These results indicate that living in the northern sector of China tend to be more of a risk to some dyslipidemias. The reasons for these associations might be partly attributable to factors such as cold temperature and dietary habits [20]. Some parts of the northern territories of China experience the cold temperate monsoon climate. This extremely cold weather could lead inhabitants to eat more animal foods as compared to fruits and vegetables due to shortages. Additionally, this cold weather can restrain people from doing sufficient outdoor physical activity during periods like the winter season. As a result of the decrease in physical activities, residents could be at risk of overweight or obesity with consequent high risk of dyslipidemia. The stroke belt zone was positively related to raised TC, low HDL-C and high TG in our study. The INTERSTROKE study observed that China’s stroke burden can be attributable to the combined effects of modifiable risk factors including metabolic risk factors such as high TC [46]. Again, the clustering of CVD risk factors among residents of northern china could partially explain this finding [47]. Therefore, the need to scale-up awareness creation and the greater use of lipid-lowering drugs is pertinent.

Previous studies have noted increasing values for BMI and WC among dyslipidemic subjects [22, 27, 31]. These findings were confirmed by our study which demonstrated that adults with higher than normal body weight (i.e., overweight/obesity) and WC (i.e., central obesity) had higher likelihoods of acquiring all dyslipidemias. Particularly, some studies have demonstrated that overweight and obesity could be related to low HDL-C and elevated TG [23, 30], and weight loss improves atherogenic lipid profile [11]. However, a longer-term perspective that focuses on controlling obesity is needed, and for this reason, considerable resources should be allocated to the promotion of healthy lifestyles to reduce obesity. Educating adults on lifestyle modification and encouraging healthy lifelong habits should be a prime focus.

According to the Framingham Heart Study, over 80% of subjects with hypertension had at least an additional disease risk factor. Hypertension and dyslipidemia are key risk factors of CVD. Studies have found that dyslipidemia can cause endothelial damage which can lead to loss of physiological vasomotor activity with consequent elevated systemic blood pressure. Also, the presence of dyslipidemia has been found to be more frequent in hypertensive than in normotensive subjects [48].

Other studies have also indicated that raised TC and hypertension can coexist, and can result in what is known as dyslipidemic hypertension [49]. Participants with raised TC, raised LDL-C, raised TG and raised non-HDL-C were more likely to have hypertension; this is comparable to other studies [23, 27]. Similarly, not only did Cetin et al. [43] demonstrate a positive correlation between raised (TC, LDL-C and TG) and hypertension, but they also reported the combined effects of hypertension and dyslipidemia on the greater likelihood of cardiovascular events. Consequently, the risk of acquiring CVD owing to dyslipidemia and hypertension co-existence could be multiplicative [37]. Appropriate management of this comorbidity will remain crucial to minimizing disease complications in the short- and medium-term.

Subjects with diabetes demonstrated higher odds of all dyslipidemia components. Diabetes-dyslipidemia comorbidity has been reported in large community-based surveys [23, 27, 41]. A previous study showed that the likelihood of cardiovascular events is increased in individuals with type-2 diabetes, and dyslipidemia could be a contributing factor with often poor results [50]. The above co-morbidities constitute the important components of metabolic syndrome (MS). Therefore, enhancing routine risk assessments and aggressive pharmacological management of dyslipidemia among affected individuals is imperative.

Our analysis revealed that current alcohol drinkers were less likely to develop low HDL-C than non-drinkers. The NCEP ATP III guideline had indicated that small amounts of alcohol could increase HDL-C levels [23]. HDL-C relates to alcohol linearly, which is homogeneous within a population and it is hardly dependent on factors of genetic origin [51]. Current alcohol users showed higher positive association with raised TC than raised (LDL-C, TG and non-HDL-C) values. Alcohol use has been found to be independently associated with raised TG in a working population [49], as well as its association with the inability to achieve adequate control of hypertriglyceridemia [52].

Our results suggest that participants who engaged in regular physical activities had lower odds of dyslipidemia. The positive effects of exercises on serum lipids have been studied extensively. Physical fitness and regular exercises can considerably reduce absolute cardiovascular risk and death rate [8, 53]. Thus, strategies to improve the adoption and adherence to exercise programs should be emphasized to improve health.

Ultimately, the high prevalence of dyslipidemia with the associated risk factors seen in this study should serve as enough incentives for steps to be taken by authorities for their control. All risk factors that could play major roles in blood lipid pathology must be targeted.

#### Strengths

This study is the first to use a nationally representative sample of middle-aged and older Chinese adults (≥40 years) to compare rural and urban prevalence of dyslipidemia and risk factors based on the 2014 CNSSPP data with a robust methodology. The geographically large and diverse coverage makes the survey generalizable for China. This study demonstrated that identified risk factors of dyslipidemia such as female sex, overweight, obesity, central obesity and diabetes were comparable to those found elsewhere. Therefore, interventions such awareness creation programs and greater use of lipid-lowering drugs [45] used in other countries could be replicated in China. This study’s results could also be used as part of China’s rural-urban dyslipidemia epidemiology baseline data. Finally, it brought to the fore the need to intensify dyslipidemia intervention programs among Chinese adults.

#### Limitations

The limitations of this study also warrant discussion. Firstly, we performed a cross-sectional study using CNSSPP data to provide a snapshot of rural and urban dyslipidemia prevalence in China, thus, causal pathways underlying the reported associations cannot be ascertained. Secondly, information on lipid-lowering therapy were not used due to missing data, which might lead to an underestimation of the prevalence, and the associations towards the null value**.** Thirdly, the likelihood of lipid lowering drugs usage among some participants could have contributed to the low prevalence of raised LDL-C in the study [54]. Fourthly, all study participants were aged ≥40, therefore the results may not be generalizable to younger populations. Lastly, information bias might have been introduced from the questions on self-reported demographic and lifestyle risk factors such as smoking.

## Conclusions

In summary, our study estimated the overall prevalence of dyslipidemia among rural and urban adults in China as 43.2 and 43.3%, respectively**.** Dyslipidemia was more common in women in both rural and urban areas. It was higher in men than in women before age 50, but increased in women than in men after age 59. Low HDL-C was higher among urban compared to rural residents with 20.8% vs. 19.2%, respectively. Raised non-HDL-C was slightly higher in rural versus urban subjects (10.9% vs. 10.0%). Raised TC and raised LDL-C values were more prevalent among rural inhabitants. Raised TG was high and similar in both rural and urban areas (22.4% vs. 22.5%). Overweight, obesity, central obesity and diabetes were common risk factors for dyslipidemias. Hypertensives and current drinkers were less likely to get low HDL-C. Therefore, in order to reduce the prevalence of dyslipidemia, a multifaceted approach with special attention to controlling identified risk factors is crucial.

## Data Availability

The dataset of the study is available from the corresponding author on reasonable request.

## Abbreviations

CHD: Coronary heart disease
CNSSPP: China National Stroke Screening and Prevention Project
CVD: Cardiovascular disease
DALYs: disability-adjusted life-years
HDL-C: Low high-density lipoprotein
LDL-C: Raised low-density lipoprotein
NCEP: National Cholesterol Education Program
Non-HDL-C: Raised non-high-density lipoprotein
TC: Raised total cholesterol
TG: Raised triglycerides
